# Models of Dynamic Belief Updating in Psychosis—A Review Across Different Computational Approaches

**DOI:** 10.3389/fpsyt.2022.814111

**Published:** 2022-04-12

**Authors:** Teresa Katthagen, Sophie Fromm, Lara Wieland, Florian Schlagenhauf

**Affiliations:** ^1^Department of Psychiatry and Neurosciences, CCM, Charité – Universitätsmedizin Berlin, Corporate Member of Freie Universität Berlin, Humboldt-Universität zu Berlin and Berlin Institute of Health, Berlin, Germany; ^2^Einstein Center for Neurosciences, Charité – Universitätsmedizin Berlin, Corporate Member of Freie Universität Berlin, Humboldt-Universität zu Berlin and Berlin Institute of Health, Berlin, Germany; ^3^Bernstein Center for Computational Neuroscience, Berlin, Germany; ^4^NeuroCure Clinical Research Center, Charité – Universitätsmedizin Berlin, Corporate Member of Freie Universität Berlin, Humboldt-Universität zu Berlin and Berlin Institute of Health, Berlin, Germany

**Keywords:** computational psychiatry, Bayesian learning, Hierarchical Gaussian Filter, change point detection, schizophrenia, psychosis, belief updating, reinforcement learning

## Abstract

To understand the dysfunctional mechanisms underlying maladaptive reasoning of psychosis, computational models of decision making have widely been applied over the past decade. Thereby, a particular focus has been on the degree to which beliefs are updated based on new evidence, expressed by the learning rate in computational models. Higher order beliefs about the stability of the environment can determine the attribution of meaningfulness to events that deviate from existing beliefs by interpreting these either as noise or as true systematic changes (volatility). Both, the inappropriate downplaying of important changes as noise (belief update too low) as well as the overly flexible adaptation to random events (belief update too high) were theoretically and empirically linked to symptoms of psychosis. Whereas models with fixed learning rates fail to adjust learning in reaction to dynamic changes, increasingly complex learning models have been adopted in samples with clinical and subclinical psychosis lately. These ranged from advanced reinforcement learning models, over fully Bayesian belief updating models to approximations of fully Bayesian models with hierarchical learning or change point detection algorithms. It remains difficult to draw comparisons across findings of learning alterations in psychosis modeled by different approaches e.g., the Hierarchical Gaussian Filter and change point detection. Therefore, this review aims to summarize and compare computational definitions and findings of dynamic belief updating without perceptual ambiguity in (sub)clinical psychosis across these different mathematical approaches. There was strong heterogeneity in tasks and samples. Overall, individuals with schizophrenia and delusion-proneness showed lower behavioral performance linked to failed differentiation between uninformative noise and environmental change. This was indicated by increased belief updating and an overestimation of volatility, which was associated with cognitive deficits. Correlational evidence for computational mechanisms and positive symptoms is still sparse and might diverge from the group finding of instable beliefs. Based on the reviewed studies, we highlight some aspects to be considered to advance the field with regard to task design, modeling approach, and inclusion of participants across the psychosis spectrum. Taken together, our review shows that computational psychiatry offers powerful tools to advance our mechanistic insights into the cognitive anatomy of psychotic experiences.

## Introduction

The computational psychiatry approach aims at explaining psychiatric symptoms via disruptions of mechanisms that underlie information processing (1, 2). How these mechanisms work (or: how information is processed) is described in computational models encompassing the equations that link the processing of sensory inputs to behavior. These generative models try to shed light on the “black box” of the mind. They serve as our dynamic representation of the changing environments that we aim to predict in order to adapt our behavior. Therefore, our *beliefs* about the world are constantly tested against sensory evidence and updated when our predictions have failed us (causing a prediction error = input - belief).

Inherent to psychotic symptoms is the mismatch between the internal beliefs about the world and the “true” environment, as in hallucinations reflecting perceptions without sensory inputs or delusions reflecting false beliefs about the world held with strong conviction despite contradictory evidence. Thus, the tools of computational psychiatry have been of high relevance to psychosis research over the past decade. It has been proposed that the altered integration of new evidence to update beliefs is at the core of psychotic experience (3, 4). As such, spurious evidence may be regarded as meaningful leading to a state of aberrant salience (5–8). For instance, I may assume that the neighbors are on to me (belief) based on the experience that they once did not greet me (sensory evidence). Moreover, certain beliefs may be held with abnormal conviction despite contradictory evidence as in delusional ideations. In our example, I may be convinced that the neighbors are wiretapping me (belief) though I have not found any bugs after rigorous searching (evidence). Both phenomena, updating beliefs too much or too little, can be computationally described via the concepts of precision and volatility. When the environment is changing (high volatility), it is very useful to consider any input that deviates from my beliefs (prediction error) as important and precise evidence. In computational terms, the learning rate applied to such a prediction error should be high in order to update the belief (new belief = old belief + high learning rate ^*^ prediction error). In contrast, when the environment is stable (low volatility), it is adaptive to keep sensory precision of deviating and noisy inputs low and beliefs should not be updated (low learning rate). Notably, our examples show that the estimation of precision and volatility in psychosis might be altered in both directions possibly depending on the stage of the disorder [i.e. delusional mood vs. chronic delusions, see (9)]. With regard to symptom domains, these higher-level learning deficits, i.e. inference about the hidden state of the environment being either noisy or truly changing, were proposed to specifically affect delusions (10, 11). However, such deficit may also cause the formation of negative symptoms via decreased motivational values being acquired during reinforcement learning (4).

For successful dynamic belief updating, learning rates must be adapted to the noisiness and volatility of the environment. Both things should usually be indirectly inferred and dissociated from each other by interacting with the environment via trial and error. Or put differently, higher-order learning requires learning when to learn and this process needs to be implemented in a dynamic learning model. There are different modeling frameworks taking on this challenging approach in different ways. As formulated by Pearce and Hall (12), learning rates or “associability” decreases with time and increases with the absolute size of previous prediction errors. In contrast to such error-based models, Hierarchical Hidden Markov Models (HMM) are fully Bayesian and instead of updating beliefs, the probabilities of different “states” (my neighbors are suspicious vs. my neighbors are friendly) are tracked and weighted against each other [for an example implementation, see (13)]. There are two relatively recent modeling frameworks, that function as error-update approximations to a fully (and therefore, costly) Bayesian learner: the Hierarchical Gaussian Filter [HGF; (14, 15)] and the change point detection modeling [CPD; (16)]. In the HGF, learning takes place in a hierarchy where the upper-level belief reflects the estimated volatility that is informed by lower-level (sensory) prediction errors. The equivalent to a learning rate is the precision weight, where upper- and lower-level precisions are weighted against each other and therefore act as a “volatility vs. noise” trade off. Whereas the HGF was designed to learn about continuous changes in the environment, change point detection theory models learning about discrete changes. Here, the assumed hazard rate (how often the environment is thought to change) informs the probability of a change point that is weighted against the relative uncertainty (how little we know) and the estimated noise in the environment.

Though these models have been applied to investigate the same question, namely dynamic belief updating in psychosis, the different modeling environments and terminologies (e.g. volatility belief vs. change point probability) have often impeded the comparability of results. They differ in their number of free parameters, their fitting procedures and their experimental tasks they are applied to. At least in parts, this heterogeneity is owed to computational psychiatry still being a relatively young field that has yet to find and agree on a methodological gold standard. Though, there are promising suggestions for modeling standards (17), such as parameter recovery or model selection studies checking all these criteria *and* involving clinical data are still warranted. This lack of a gold standard calls for more technical considerations about the modeling that should be taken into account when interpreting the results of dynamic belief updating in psychosis especially across studies.

Here, we review dynamic learning models and their respective behavioral findings in subclinical and clinical populations with psychotic experiences. Thereby, we focus on the two influential dynamic learning models, the HGF and change point detection theory. We performed a literature search for publications citing a relevant paper from both modeling approaches (14, 16) and reporting results in participants from the psychosis spectrum. For a more comprehensive review, we extended our scope to other selected modeling approaches, such as, among others, extensions of Pearce-Hall learning (12) and Hidden Markov Models (13, 18). For the scope of this review, we are focusing on the potentially impaired learning about changing environments and its relation to the continuum of schizophrenia symptoms. Thus, we restricted our review to learning tasks with dynamic environments and/or noisy inputs (see Table 1) and excluded paradigms explicitly manipulating perceptual uncertainty. We will briefly touch upon the latter phenomenon in our discussion section, but would refer to a more in depth review on the role of priors and (ambiguous) sensory evidence in perception as a model for hallucinations (10). In the following, we will individually describe the core foundations of these different modeling approaches together with a summary of the respective results on psychosis. In our discussion, we will interpret the similarities and differences in behavioral findings across the different approaches while minding the diverse modeling backgrounds. Our aim is to draw conclusions about dynamic belief updating in psychosis and highlight the difficulties and potentials of computational modeling of behavior, also regarding future computational psychiatry studies.

**Table 1:** Overview of the selected studies with their respective tasks and modeling details.

**First author**	**Year**	**Sample**	**Task**	**(Best) Model**	**Simulation (1) of raw results/ for (2) parameter recovery**	**Computational parameters or trajectories altered in psychosis and/ or schizophrenia**		Model com-parison^*^	**Input / behavioral response**
						**Group differences**	**Symptom correlations**		
Adams et al. (19)	2018	79 PSZ or delusional P, 22 nonpsychotic mood disorders, 146 HC	Probability estimates beads task with trial-wise confidence and probability rating;	HGF2 with evolution rate, initial variance, belief instability and response stochasticity	no / yes	In clinical psychosis: higher κ1 (stronger belief update following disconfirming events) and lower v (inverse decision noise)	v correlated with higher IQ in PSZ	yes	binary / continuous
Katthagen et al. (20)	2018	42 PSZ,42 HC	Implicit Salience Paradigm: outcome detection following cues with relevant and irrelevant features for outcome prediction; reversals for reinforcement and relevance (160 trials);	HGF2-Relevance weighted prediction error and irrelevance bias and w/o Precision Feedback;	yes / no	Bias to irrelevant information (mean β_irrel) increased in PSZ	β_irrel correlated with increased negative symptoms	yes	binary / continuous (reaction time)
Cole et al. (21)	2020	13 CHR (antipsychotic-naïve), 13 HC	RLT with stable and volatile phases (160 trials)	Autoregressive HGF3-DU-V	no / yes	Higher m3 in CHR, group-by-phase interaction on μ3 trajectory with larger increase of μ3 after first reversal in CHR	/	yes	binary / binary
Deserno et al. (22)	2020	70 PSZ, 64 HC	RLT with stable and volatile phases (160 trials)	HGF3-DU-V	yes / no	heightened initial μ3 and κ in PSZ	Initial μ3 correlated with lower executive functioning and lower cognitive speed	yes	binary / binary
Diaconescu et al. (23)	2020	70 HP, 81 LP (subclinical)	Advice-taking experimental paradigm with two framings (between-subjects: situational vs. dispositional) with stable and volatile phases (210 trials);	HGF3-V-integrated advice taking	no / no	Overall: less pronounced framing effects in HP; Parameters: ω2 and ζ differed less across task frames in HP (interaction with frame); Precision trajectories: only in LP lower precision belief in dispositional vs. situational frame; impact of volatility was stronger in the situational compared to the dispositional frame only in LP;	/	yes	binary / binary
Henco et al. (24)	2020	31 HC, 28 MDD, 29 PSZ, 28 BPD	RLT with parallel social/non-social cues (120 trials)	Autoregressive HGF3-DU-V (both cues)	yes / yes	Higher ζ (weighing social over non-social info) in PSZ (and BPD) compared to HC	/	yes	binary / binary
Reed et al. (25)	2020	27 HP, 77 LP (clinical and non-clinical)	RLT with 3 options, fixed and adaptive reversals (160 trials)	Autoregressive HGF3-DU-V	yes / yes	Higher initial μ3 and κ, only online sample: lower ω	κ (Block 1) positively correlated with paranoia, depression and anxiety	no	binary / categorical (3 options)
Suthaharan et al. (26)	2021	193 HP, 793 LP	RLT with 3 (social or nonsocial) options, fixed and adaptive reversals (160 trials, see Reed et al.)	Autoregressive HGF3-DU-V	no / no	HP: lower ϑ, higher initial μ3, higher κ, lower ω	initial μ3 correlated with more conspiracy and anti-vaccine beliefs	no	binary / categorical (3 options)
Kaplan et al. (27)	2016	17 PSZ, 24 HC, 35 unaffected siblings of PSZ	Dynamic numerical inference task (320 trials)	Normative reduced Bayesian change point detection model	no / no	Higher estimated posterior probabilities of change point in PSZ vs. HC	/	no	continuous/ binary
Nassar et al. (28)	2021	94 PSZ, 33 HC	Helicopter location inference task: Position of helicopter must be inferred in change point or drifting oddball conditions, with appetitive and non-appetitive framing (400 trials)	Normative reduced Bayesian change point detection model with perseveration + 2 context error terms	yes / yes	Behavior of PSZ not better explained by normative model with high hazard rate	Perseveration factor negatively related to cognition in PSZ but not HC	yes	continuous / continuous
Schlagenhauf et al. (13)	2014	24 PSZ (unmedicated), 24 HC	Probabilistic RLT (200 trials)	HMM (R/P) for 22/24 HC but only 13/24 PSZ	yes / no	Reward sensitivity differed between HC and poor-fit PSZ but not HC and good-fit PSZ; overestimation of transition rate in PSZ.	Model fit of HMM correlated with lower positive symptoms in PSZ	yes	binary / binary
Vinckier et al. (29)	2015	21 HC (under Ketamine and Placebo)	RLT with stable task phases, 3 fixed reversals (240 trials)	Hierarchical learning with double-update, where c scales the (nondifferential) effect of confidence on α and β; confidence relies on choice-optimality; reinforce = outcome sign;	no / no	Ketamine reduced confidence-weight on learning rate α and softmax temperature β	/	yes	binary / binary
Baker et al. (11)	2019	24 PSZ 21 HC	Incentivized information-sampling task (modified version of the beads task with varying ratios, 60:40, 75:25, 90:10, 100:0)	Bayesian inference with weights for recency bias on priors and sensory likelihood weight	yes / yes	Decreased information seeking in PSZ when adjusting for delusion severity, but this was driven by socio-economic status	Prior-weight ω1 affected slower updating and correlated with higher PDI scores (total and subscores), as well as with suspiciousness/persecution (PANSS-P6)	yes	binary / continuous
Haarsma et al. (34)	2020	24 ARMS 20 FEP 30 HC	Predicting rewards drawn from distributions with fixed means and cued high or low precision (186 trials)	Pearce Hall model with precision adaptation and separate parameters for signed and unsigned prediction errors	yes / no	For FEP best fit of RW without precision weighting; HC and ARMS participants show higher α in the high-precision condition, FEP do not	/	yes	continuous / continuous

*Hierarchical Gaussian Filter models in green, Change point detection modeling in yellow, Selection of various other dynamic models in grey. ARMS, At Risk Mental State; CHR, Clinical High Risk; FEP, First Episode Patients; HC, Healthy Controls; HP, High Paranoia; LP, Low Paranoia; PSZ, Patients with Schizophrenia; RLT, Reversal Learning Task; DU, Double Update; HGF, Hierarchical Gaussian Filter; HMM, Hidden Markov Model; RW, Rescorla-Wagner; V, Volatility; PANSS, Positive and Negative Syndrome Scale; PDI, Peters Delusion Inventory; Computational parameters or trajectories: α, learning rate; β, softmax temperature; δ, prediction error; k, 2^nd^ and 3^rd^ level coupling (HGF models); μ3, dynamic volatility belief; ω, constant component of 2^nd^ level belief update/evolution rate; ϑ, meta-volatility; m3; mean-reversion equilibrium;*

* *Model selection in terms of formal comparison between quantitative model fit indices*.

## The Hierarchical Gaussian Filter

Based on our literature review, the HGF clearly outweighs other model types by the number of applications in psychosis studies. We found ten articles that used the HGF for modeling the learning process in changing and/or noisy environments of participants from the psychosis spectrum.

### The Practical Side

The HGF models are implemented in the tapas toolbox by the Translational Neuromodeling Unit (TNU) project, which is therefore easily accessible via a free download. Another practical advantage lies in the relatively easy adjustment of these existing models to individual task data via the combination of “perceptual or learning models” (generating the dynamic learning trajectories, like prediction errors and beliefs, e.g. HGF or Rescorla-Wagner) and “decision or response models” (how learning trajectories translate into behavior, e.g. softmax). Thereby, individual task-based adjustments are usually done in the response models whereas the often more complex “learning models” can remain (relatively) stable across task applications.

### The Theory and Key Parameters

The HGF is a generative model mapping prior beliefs about the changing hidden states of the world to sequentially appearing sensory evidence. Beliefs span multiple (but usually three) levels and are continuously evolving in Gaussian random walks. The belief update combines Bayesian principles (weighting prior beliefs against sensory evidence according to their respective probability distribution, i.e. precision/inverse uncertainty) with the biologically plausible framework of hierarchical prediction errors. The three levels describe different kinds of beliefs about the state of the world. The first level describes μ_1_ the belief about the state of the sensory input. This level is only relevant when there is perceptual ambiguity (outcome uncertainty σ_1_) about the input; otherwise and as in tasks with binary inputs like rewards and losses, this is the sigmoid transformation of the second level belief. On the second level, the belief μ_2_ reflects the tendency x_2_ (or probabilistic strength) of a state x_1_ being true (e.g., stimulus A leads to reward) and is associated with expected uncertainty σ_2_ (uncertainty that can be predicted by the model, e.g. highest when this tendency is close to 0.5). On the third and last level (though the HGF framework would allow for additional levels), the belief about the environmental volatility (μ_3_) is tracked. Crucially, the higher-level beliefs work downstream by determining the impact of lower-level prediction errors on changing the beliefs. The core algorithm lies in the definition of precision weights that are used equivalent to dynamic learning rates, weighing the influence of prediction errors on updates (Equation 1).


(1) $$\begin{array}{r} \frac{{\hat{\pi }}_{1}}{\pi_{2}}\;=\;\frac{{\hat{\pi }}_{1}}{{\hat{\pi }}_{2}\;+\;{\hat{\pi }}_{1}}\;=\;\frac{{\hat{\pi }}_{1}}{\frac{1}{\sigma_{2}^{\left(t\;-\;1\right)}\;+\;exp\left(\kappa \mu_{3}^{\left(t\;-\;1\right)}\;+\;\omega_{2}\right)}\;+\;{\hat{\pi }}_{1}} \end{array}$$

Those precision weights consist of the lower-level predicted precision ${\hat{\pi }}_{1}$ in the numerator and the same-level precision π_2_in the denominator. In a three-level HGF, the 2nd level's precision contains the higher-level belief μ_3_, which reflects the volatility belief, as well as the expected uncertainty σ_2_. Thereby, learning rates are high when the environment is thought to be volatile and changing (as reflected in high values of μ_3_) and thus prediction errors are weighted more strongly. In contrast, when the environment is thought be stable or when there is low expected uncertainty, learning rates are low and prediction errors are “explained away” as noise.

**Figure 1:**
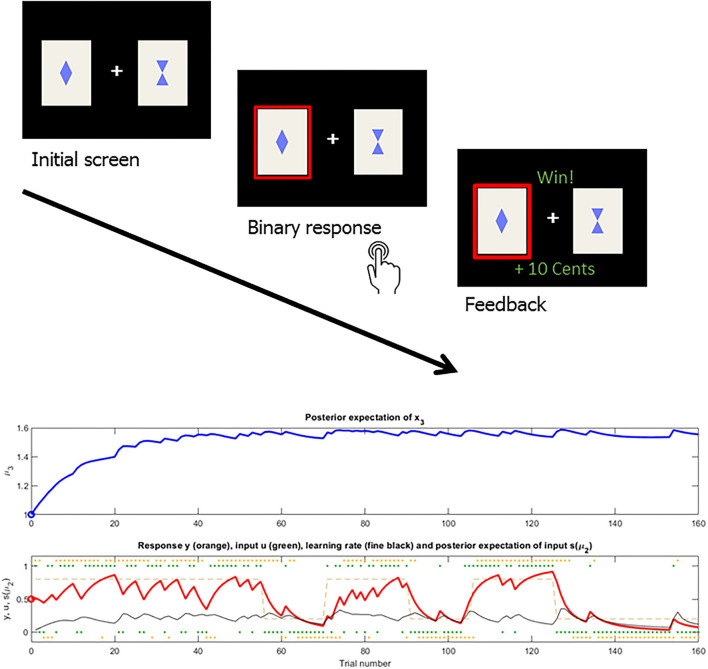
Reversal learning task example and the associated HGF learning trajectories fitted to binary choice data of an individual participant. Upper plot: Depiction of trial sequence in a volatile reversal learning task with geometric stimuli (rewarded in this trial) [as in (22)]. Participants had to make a binary choice between one out of two stimuli via a button press and were presented with either a reward or loss outcome. Lower plot: Upper panel: Blue line represents one subject's individual trajectory of higher-level belief μ_3_ over the course of the task from trial 1–160. Lower panel: Underlying contingencies are depicted in light brown with anti-correlated reward probabilities of one of the stimuli, reward contingencies remain stable in the beginning and end of the task with a volatile reversal period in between. Red line represents the belief μ_2_ and reflects the tendency x_2_ (or probabilistic strength) of stimulus A leading to reward. Black line represents the dynamic learning rate on the second level belief μ_2_.

Among the free model parameters to be fitted individually, there is usually ω_2_, the step-size of the Gaussian random walk on the second level, or put a bit simpler, the fixed proportion of the dynamic learning rate. There is κ for coupling the influence of the third level's belief on the second level. When this parameter is fixed to 0, the HGF reduces to a two-level model. The ϑ or ω_3_ parameter (the same parameter is meant, the letter only depends on the toolbox version) reflects the meta-volatility and determines the step-size of updates in the beliefs about volatility. In a modified version of the HGF (21, 23) the third-level belief also contains a “reverting parameter” *m*, reflecting a value where the volatility belief is steadily being pulled back to, in order to prevent strong drifts. Apart from these parameters, starting points for the dynamic belief and uncertainty trajectories ($\mu_{3}^{0}$ and $\sigma_{2}^{0}$) can also be fitted to individual behavior. For example trajectories of 2nd and 3rd level beliefs fitted during a probabilistic reversal learning task, see Figure 1. Besides the strong potential of uncovering dysfunctional mechanisms via the variety of free parameters, it should be noted that sufficient parameter recovery as a basis to make valid interpretations was not given for all parameters, especially the meta-volatility ϑ (21, 35). Further, as the HGF assumes learning via continuous Gaussian random walks, simulations have shown that when applied to fast changing paradigms, i.e. in changing states as in reversal learning, it seems inferior to the CPD (36) or the Volatile Kalman Filter (37).

### HGF: Psychosis Findings

In the context of psychosis, the HGF was applied to behavioral responses of the following paradigm types: reversal learning (21, 22, 25, 26), social advice taking (23, 24), an implicit salience paradigm (20), and a modified version of the beads task (19).

For volatile reversal learning (as depicted in Figure 1), subjects have to choose between neutral stimuli in order to find out the better option, i.e. the stimulus that has the highest probability of being rewarded or leading to the most points. Each choice is followed by feedback, given either by monetary rewards/losses (22) or by points (21, 25, 26). Critically, the “ground truth” contingencies change over time, by either reversing (i.e., the better stimulus becomes worse, such as 0.8 to 0.2), while the previously less valuable stimulus is reinforced more often (0.2 to 0.8) and/or a change in the probabilities (i.e., probability of 0.9 reinforcement lowering to 0.6). In the studies by Cole et al. (21) and Deserno et al. (22), volatility manipulation only took place in terms of the number of performance-independent reversals while otherwise keeping stable probabilistic contingencies (0.8/0.2). In the studies by Suthaharan et al. (26) and Reed et al. (25), such reversals did not only appear at fixed time points but also in line with task performance, i.e. after having chosen the better option in 9 out 10 trials. Crucially, there were also unsignaled contingency transitions. In the beginning, the three stimuli had 90, 50, and 10% reinforcement rates and halfway through the task, those probabilities changed to 80, 40, 20%. Despite these differences in task design and clinical characteristics, results have been rather consistent across studies. Patients with manifest psychosis showed lower overall performance and more switching between choices (22, 25, 26), while no “raw” performance data were reported for the study in clinical high risk participants (CHR) (21). Parameters determining the “basic level” of volatility, either as the starting point of the belief trajectory or the reverting value of this belief, were increased in patients with schizophrenia (22), clinical high-risk subjects (21) and in subjects with high (mostly subclinical) paranoia (25, 26). Further, the influence of this higher-level volatility belief on learning about the lower-level association strength, captured in the κ parameter, was stronger in patients with schizophrenia and high paranoia (22, 25, 26). However, this difference in the kappa parameter was absent in CHR (21).

Most of these computational studies on psychosis investigated altered learning as a “content-free” cognitive process that can potentially be generalized across contexts. However, especially delusions are often characterized by a strong social component, such as the typical assumption that others are hiding their harmful intentions. Some studies have investigated this by introducing social aspects into learning paradigms with ranging complexity: from “classic” reversal learning between different social avatars (26), over integrating the associative strengths with rewards over social and non-social cues (24) to a combination of the latter with context-manipulations via cover-stories (23). One study comparing the use of social avatars (emojis) to non-social stimuli (color of card-decks) for reversal learning in participants with subclinical (and still very low) paranoia, did not find any differential effects on the learning process, neither in raw data nor in computational analyses, though the stimuli were perceived as sabotaging by the delusion-prone group (26). In a reversal task, where social (gaze direction) and non-social information (card deck) had to be integrated simultaneously, patients with schizophrenia performed overall worse and relied more on the social compared to the non-social information (24). The latter was reflected in a response model parameter weighting social over non-social beliefs in the decision process. Here, both cue types had independent associative strengths that varied over the course of the experiment and thus required parallel volatility-dependent learning which was captured in separate 3-level-HGFs for parallel learning about two cues comparable to the perceptual models in (20). Further, there was no instruction “personalizing” the social cue as representing an individual with (hidden) intentions. This was different in the study by Diaconescu et al. (23), that investigated social inference (i.e., about the intention of others) in subclinical paranoia. Subjects had to predict the outcome of a binary lottery and received social (video of an adviser) and non-social (pie chart) advice. Notably, the adviser was either introduced to give correct or false advice due to either task structure (situational) vs. acting based on his intentions (dispositional). Unlike in the otherwise very similar study by Henco et al. (24), nothing could be learned about the non-social cue representing the true reward probability. Hence, computational modeling was only used to infer the subject's beliefs about the adviser's validity which was volatile across the task. Whereas both groups applied hierarchical learning, only low paranoia subjects were susceptible to the task framing. When the adviser's intentions were highlighted, they took social advice less into account (higher weighting of non-social information) and updated their belief about the adviser's validity less rapidly (lower ω_2_ learning parameter). In contrast, high paranoia subjects did not differentiate between framings [for raw data results, see (38)]. Further, volatility affected precision beliefs more in the situational than in the dispositional framing, but this effect was only present in low paranoia. Though there was no main effect of group here, the decreased susceptibility to social framings seems a bit at odds with the finding that patients with schizophrenia follow social cues more than the simultaneously presented, non-social ones (24).

It has long been proposed that schizophrenia patients have trouble down-regulating irrelevant information (5–8). Transferring this to belief updating, irrelevant stimuli may elicit learning signals in the brain causing the formation of aberrant associations (39, 40) and blurred dissociation between relevant and irrelevant events (4). In line with this, the HGF was applied to modeling biased belief updating toward irrelevant (20) and disconfirming evidence (19). For investigating the bias toward irrelevance in an outcome detection task, preceding cues varied in two dimensions whereby only one of them was relevant for predicting the outcome and these predictive value changed over time (20). Reaction times to the outcomes were best described by a 2-level-HGF defining subjective relevance based on the first level precision π_1_. The best model further contained a bias to irrelevant information which was heightened in patients with schizophrenia exhibiting more negative symptoms. Adams et al. (19) investigated biased belief updating with a modified beads task, where patients had to infer the underlying urn of ongoing draws of beads. As the environment was stable (no underlying changes in urns), a 2-level HGF was applied to model choice behavior. The parameter κ_1_ [in former studies used for the coupling between levels 2 and 3 as in Equation (1), e.g. in (22)] now played a key role in coupling levels 1 and 2 in the sigmoid transformation of the second-level belief for the behaviorally relevant first-level belief; higher κ_1_values caused stronger updating following disconfirming events and lower updating following consistent draws. Besides higher levels of decision noise, this κ_1_ parameter was also increased in patients with schizophrenia interpreted as a bias to more drastic belief updating toward disconfirming evidence.

## Change Point Detection Theory

We identified two papers that modeled dynamic learning in psychosis using change point detection algorithms (27, 28). The centerpiece of change point algorithms is a “normative model” derived from a Bayesian ideal observer that describes optimal learning behavior (16). The approach has also been applied in other patient populations (41).

### The Practical Side

Resources of change point detection algorithms are not systematically documented in a toolbox, but code (mostly in Matlab) is freely available from repositories of the seminal authors (https://sites.brown.edu/mattlab/resources/). As described for the HGF, inter-individual differences in behavior can be captured via fitting of free model parameters (27). Alternatively, and more often used for this approach, participants' adherence to the normative model with optimal computational learning parameters is used in combination with linear regression. In these models, regression coefficients capture whether the trial-wise update can be predicted by the normative learning parameters (42, 43). Further, different model predictions can be computed by altering specific parameters of the normative model in line with learning aberrations that are hypothesized to be altered in psychosis.

### The Theory and Key Parameters

Volatile environments are characterized by fluctuating action-outcome contingencies. In such environments, beliefs are required not to evolve gradually but shift discretely. According to change point detection theory and in line with Bayesian learning, an agent's belief can be formalized as a distribution with the mean reflecting the current prediction. Deviating inputs can be due to noise (as reflected in the variance) or due to a change in the generative distribution (mirroring environmental volatility). In other words, one infers whether the given input (X_t_) was generated by the previously learned Gaussian distribution given the current belief and noise [*N*(X_t_|Belief_t_, Noise)] or, in case of a change point, from a novel distribution. Like the HGF, change point detection modeling reflects a prediction-error-based approximation to costly optimal Bayesian learning. Beliefs are learned via prediction error-driven updates that are weighted via dynamic learning rates according to two dynamic components: (i) change point probability Ω, and (ii) uncertainty τ. According to (Equation 2), learning rates α are scaled by the current uncertainty belief when no environmental change point occurred (Ω ≅ 0). This can be overridden when a change point is detected, i.e. Ω ≅ 1, allowing a rapid elevation of the learning rate (see Equation 2).


(2) $$\begin{array}{r} \alpha^{t}\;=\;\Omega^{t}\;+\;\tau^{t}\times \;\left(1\;-\;\Omega^{t}\right) \end{array}$$

The probability of a change point (Ω) is high when the agent observed a large prediction error which signals a true change in the environment (stemming from a novel generative distribution). The agent must adopt a high learning rate to rapidly update her belief according to the new observation. Thus, Ω increases after large prediction errors so that old beliefs can be disregarded, and rapidly decreases right afterwards so that new information can be integrated. Besides prediction error magnitude, a set-level-estimate of environmental volatility (how frequent change points are in general), termed “hazard rate” feeds into this. If the agent assumes few change points (low hazard rate), she can tolerate larger prediction errors without increasing the learning rate and assuming a true change point. After a change point has occurred, the agent is highly uncertain about the new belief, represented in high relative uncertainty τ, since the new belief is based on scarce prior information. Accordingly, she adopts a high learning rate to integrate the new observation in the current belief. Intuitively, when information from the same generative distribution accumulates, beliefs become more precise, and uncertainty decreases. During such phases, the learning rate slowly decreases to allow stability of beliefs. It should be noted that previous applications of the CPD followed a rather descriptive approach by introducing central “ground truth” information of the task into the modeling process that remained unknown to the individual, as the hazard rate or uncertainty due to noise [e.g., (28, 43)]. Thereby, behavioral differences can only be normatively interpreted in terms of deviations from optimal learning, instead of explicitly modeling the interindividual differences by mechanistic parameters underlying such sub-optimal learning.

### CPD: Psychosis Findings

Change point detection algorithms were applied to data from two paradigms that required dynamic belief updating in volatile environments, including the dynamic numerical inference task (27) and the helicopter task (28). The underlying structures were very similar: continuous inputs were sampled from Gaussian distributions whose means changed discretely at several time (“change”) points. Subjects had to dynamically infer these underlying means. Thereby, they had to differentiate random noise from meaningful change points. These two components had opposing effects on learning rates. Notably, in addition to inferred prediction errors and learning rates derived via computational modeling, continuous inputs and responses allow for the calculation of directly observed (or *raw)* prediction errors and learning rates.

In the dynamic numerical inference task (27) subjects were presented with a stream of integers and indicated via button press when they assumed a change in the underlying mean. Raw behavioral prediction errors were larger in patients with schizophrenia than in controls, suggesting overall worse performance. Additionally, patients misinterpreted small prediction errors as change points that were considered noise by controls. A maximum likelihood approach was adopted to fit the model to behavioral data and estimate inferred noise and hazard rate as free parameters. While groups did not differ in these parameters, individual posteriors of change point probability were higher in patients vs. controls. Taken together, raw and modeling results indicate that patients overestimated the number of environmental change points.

**Figure 2:**
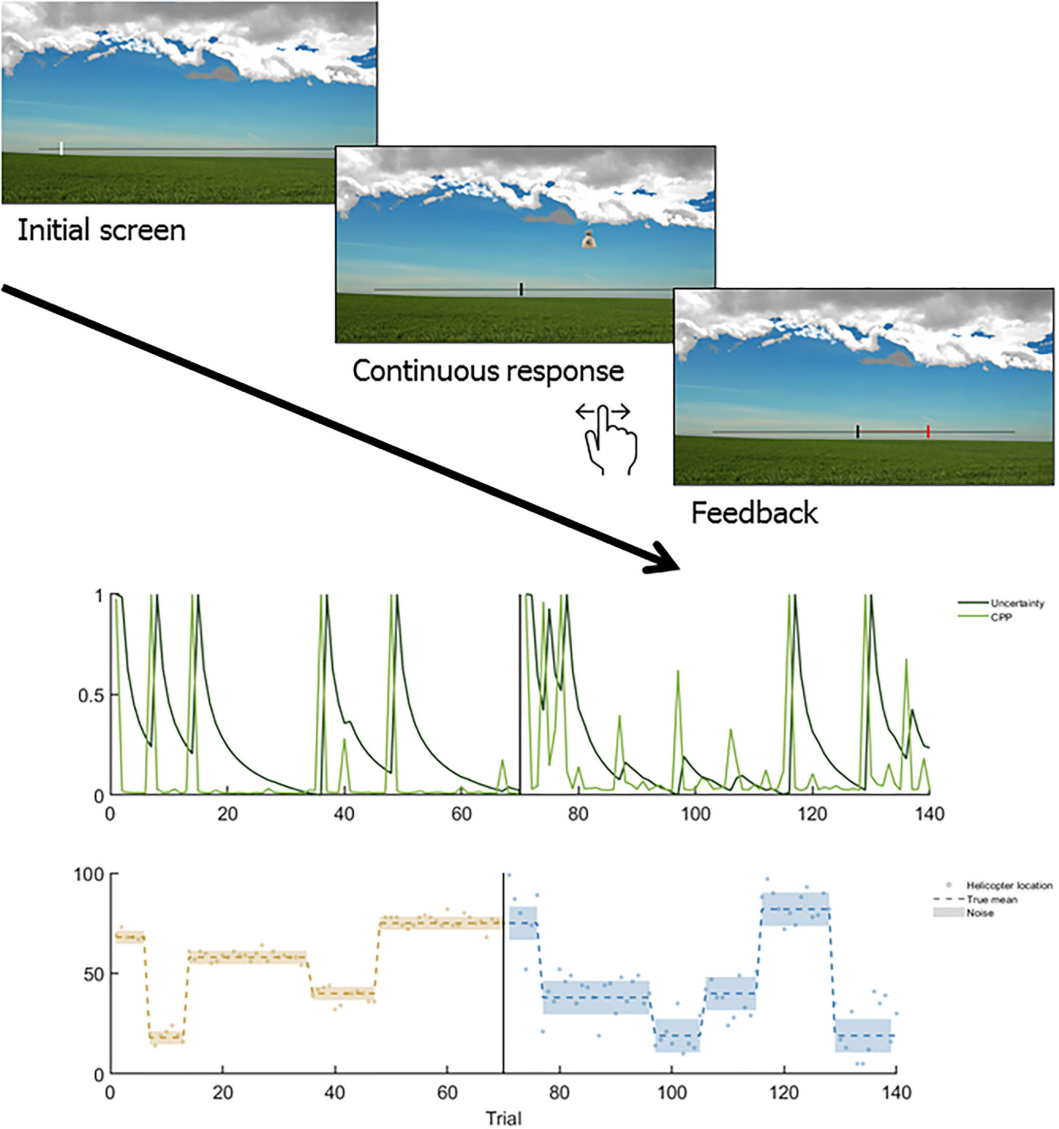
Example of the Helicopter paradigm (12) and the associated CPD learning trajectories. Upper plot: Depiction of trial sequence in the helicopter task, in which a hidden helicopter moves horizontally and drops a bucket in each trial. Participants have to give a continuous prediction of the bucket location via joystick which is followed by feedback with a visualized prediction error (the distance between their prediction and the actual bucket location in red). Middle plot: Light green represents the optimal trajectory of change-point probability and dark green represents the optimal trajectory of change-point probability (CPP) over the course of 140 trials. Lower plot: Dots represent the helicopter's locations, dispersing around the true mean (dashed line) in the low noise block (light brown) and the high noise block (light blue). Y-axis corresponds to the horizontal scale in the upper part.

In a large sample of patients with schizophrenia and schizoaffective disorder, Nassar and colleagues (28) adopted the well-established helicopter task (16, 42, 43). In this task, participants must track the location of a hidden helicopter (see Figure 2) in an uncertain and changing environment with the goal to catch bags that the helicopter drops. The helicopter stays in place for some time, but occasionally moves to an entirely different location (change point). There was also an explicitly instructed “oddball condition,” in which underlying means drifted slowly and surprising outcomes were one-off outliers that did not signal a change point. Across both conditions, patients more often used learning rates close to zero or close to one, and less often moderate learning rates. As such, they failed to integrate over prior observations which is necessary to maximize precision, but either perseverated or completely replaced their belief. Nassar et al. (28) simulated data with increased hazard rates, corresponding to an overestimation of environmental volatility. This models an agent who, when observing a large prediction error, learns too fast in the change point condition mistaking noise for change points. In the oddball condition, learning would be too slow, since updating “too much” in response to the oddballs would distract from learning about the one underlying, stable state. However, neither patients nor controls adhered to this model. Instead, Bayesian model comparison favored a variation of the normative model with free parameters for the perseveration probability. Interestingly, these parameters contributed most to a classifier for patient status, suggesting that patients perseverate more. Critically, the perseveration parameters were not related to positive or negative clinical symptoms but to overall cognitive function.

### A Selection of Other Models: Pearce-Hall, Modified Rescorla-Wagner Learning and Bayesian Inference

In this section, we will highlight a diverse range of computational studies on psychosis beyond the presented frameworks of the HGF or CPD; namely Pearce-Hall learning with precision weights (34), Rescorla-Wagner learning with different kinds of learning rate modulation (29–31), Hidden Markov models [HMM; (13, 18)] and Bayesian inference (11).

In non-hierarchical models, there is a high variety of how to computationally define dynamic learning rates. A central question is when learning should increase, for those events that are novel and thereby unreliable in predicting outcomes or for those that have proven to be reliable in the past? The models by Pearce and Hall (12) and Mackintosh (44) diverge here. Whereas Mackintosh proposed that one learns more about stimuli with ongoing occurrence, Pearce and Hall proposed that learning rates (or associability) are highest during the first occurrences of a novel stimulus {for [also more recent] hybrid solutions, please see (45–47)}. A variation of a Pearce-Hall model (8) was applied to a psychosis dataset (34). In “classic” Pearce-Hall learning, the associative strength *k* of a stimulus represents a dynamic learning rate used to update the stimulus value μ. The learning rate is affected by the opposing effects of (i) a learning decay factor γ over time (see above) and (ii) the previous absolute prediction error |δ^(*t*−1)^|. The former reflects the idea that we learn most in the beginning and then less when encountering the same stimulus-outcome pairing repeatedly. However, the learning rate can then again be upregulated to support novel learning when large prediction errors occur signaling that something has changed.


(3) $$\begin{array}{r} \mu^{\left(t\right)}\;=\;\mu^{\left(t\;-\;1\right)}\;+\;\frac{k^{\left(t\right)}\delta^{\left(t\right)}}{\omega } \end{array}$$

(4) $$\begin{array}{r} k^{\left(t\right)}\;=\;\gamma C\frac{\left|\delta^{\left(t\;-\;1\right)}\right|}{\omega }\;+\;\left(1\;-\;\gamma \right)k^{\left(t\;-\;1\right)} \end{array}$$

Diederen and Schultz (48) have applied this modeling approach to a novel learning task, where subjects had to predict the (hidden) mean of an underlying distribution. In order to investigate the effects of precision (inverse variance) scaling on prediction errors, the width of these distributions varied and was explicitly signaled to the participants. For computational modeling, the Pearce-Hall model was adapted now containing the ω*-*trajectory, that was informed by the true variance of the distributions and scaled learning rates as well as prediction errors, directly (see Equations 3 and 4). This framework was tested in a sample representing a broad psychosis spectrum: first-episode psychosis (FEP) patients, at-risk-mental-state (ARMS) individuals and healthy controls. While ARMS and healthy controls used precision-weighting and followed a Pearce-Hall learning rule, FEP patients were best fit by a simple Rescorla-Wagner model with fixed learning rates and no precision-weighting (34). More specifically, FEP generally exhibited impaired task performance because their learning was not appropriately adapted to different levels of precision.

In close relation to the Pearce-Hall model, Krugel et al. (32) proposed another formulation for learning rate modulation in error-update learning, applied to the already mentioned adaptive reversal task (25, 26). Here, the learning rate depends on the slope of averaged previous prediction errors. Thereby, with increasing absolute prediction errors, learning increases and vice versa for the opposite direction. When this task and model were administered to a sample of medicated patients with schizophrenia and controls, there were no group differences in the fitted free parameter (31). However, when comparing learning rate trajectories, patients exhibited smaller decreases in learning rates before reversals and smaller increases in learning rate after reversals. This blunted, less dynamic learning rate modulation was more pronounced in patients with high avolition.

While in the previous two models the learning rate increased with high (preceding) prediction errors, Stuke et al. (30) proposed an opposing model where learning rates are downregulated when the current prediction error is high. However, these formulations do not need to be at odds. The former models work well in unstable environments where prediction errors can signal true volatility. In contrast, when the environment is known to be stable but noisy, the mechanism proposed here works as “resilience” against irrelevant information. Computationally, the influence of large prediction errors is downregulated according to its absolute size and a resilience parameter. Using a modification of a cued beads task, subjects had to infer one out of two origin lakes (i.e. underlying distributions) of drawn fishes. They found that attenuated resilience against irrelevant information correlated with higher jumping to conclusion behavior (i.e. reaching a premature decision without gathering enough evidence experimentally reflected in reduced draws-to-decisions), as well as more delusional ideation.

Relying on Rescorla-Wagner learning about “Q-values” that reflect the expected reward for respective actions [e.g. choosing stimulus x; (49)], Vinckier et al. (29) introduced a computational definition of confidence. Confidence was operationalized as a higher-level learning trajectory affecting the lower-level learning rates and choice behavior. This trajectory reflects the subjective certainty that the chosen stimulus leads to the most optimal outcome. By its coupling with the learning rate for Q-values, in states of high confidence, value updating via disconfirming outcomes (different valence than expected) are down-regulated whereas belief confirming outcomes (same valence as value) are upregulated. In low confidence, this dissociation is “turned off” because then, all information is valuable. Further, confidence directly affects choice behavior via the coupling with decision noise; whereas high confidence leads to more exploitation, low confidence leads to more exploration, i.e. choice stochasticity. Tested in healthy controls following a ketamine challenge, this model explained choice behavior in a probabilistic reversal task. Although there were no correlations with psychotic-like symptoms, ketamine attenuated the effect of confidence on lower-level learning rates and choice behavior. The authors concluded that this psychosis-like doubt or uncertainty might explain the failure to stabilize acquired knowledge in noisy environments.

So far, the computational models presented here reflected versions of error-based belief updates with varying complexity. There, beliefs reflect Q-values of stimulus-outcome associations that are computationally represented in an array of point estimates. Another line of models is based on Bayesian learning, where posterior beliefs reflect the estimated probability of potential hypotheses (or task states) being true given current evidence. Thereby, beliefs are represented as probability distributions with their entropies reflecting the respective uncertainty. Optimal Bayesian learning requires high computational costs. Hidden Markov Models (HMM) represent a very efficient application because only the last step is needed to inform the present one and probabilities of transitions are supposed to remain stable. These transitions take place between the hidden states of the task, as in reversal learning, where the good stimulus reverses over time (e.g. state x_1_ = {*stimulus A*→*reward*; *stimulus B*→*punishment*} and state x_2_ = {*stimulus A*→*punishment*; *stimulus B*→*reward*}). Especially when tested against other, less complex models, it can reveal whether subjects only learn about *Q*-values or whether they try to infer the hidden structure of the task. Such a model comparison was carried out after modeling probabilistic reversal learning in unmedicated patients with schizophrenia and healthy controls (13). The healthy participants used their inferred knowledge of the task structure as indicated by a better model fit of the HMM. In contrast, a subgroup of patients characterized by high positive symptoms severity used simpler learning not taking into account the structure of the task. Schizophrenia patients showed impaired task performance and overestimated the probability of reversals compared to controls as indicated by higher transition rate parameters in the HMM. Nour et al. (18) employed an HMM together with a learning paradigm where healthy subjects had to infer the currently relevant cue modality (visual vs. auditory cues). This allowed them to dissociate meaningful Bayesian surprise (via applying Kullblack-Leibler divergence to the individually modeled beliefs) indicating a change in task states from meaningless sensory (information-theoretic) surprise. Free parameters indicated the cue validity reflecting the probabilistic association with rewards as well as the state transition rate for how often a change in relevance between cue modalities would occur. Whereas these free parameters did not relate to subclinical psychosis measures, overall task performance and the magnitude of belief shifts on informative compared to non-informative trials both negatively related to paranoia. In another Bayesian inference model, this time reflecting an ideal observer, Baker et al. (11) modeled behavior in an information sampling paradigm. Their self-paced version of the beads task penalized for the number of draws until decision as well as for incorrect decisions. Thus, the ideal observer has to counterbalance the costs of (future) draws increasing certainty with those costs of incorrect decision due to too low certainty. According to an HMM approach, decision making relies on Bayesian inferencing concerning the hidden states potentially underlying the evidence. When applied to the beads task this hidden state reflects the identity of the origin jar the beads might be drawn from [for a similar task design with alternate modeling, see the study by Adams et al. (19) in the HGF paragraph]. Trial-by-trial, subjects had to give explicit probability estimates on the jar's identity and those estimates were used for modeling the posterior beliefs. The best model comprised free parameters scaling the impact of prior beliefs, reflecting a primacy vs. recency bias of prior knowledge, as well as the weight of new sensory evidence on belief updates. In contrast to previous assumptions on the jumping to conclusions bias proposed to underlie psychosis (50), Baker et al. (11) observed increased draws-to-decision behavior in patients with schizophrenia, who exhibited increased delusions. On the computational level, this was explained by a stronger reliance on prior beliefs formed early in the information sampling process.

In line with the HGF and CPD, the models of this section implement the assumptions that beliefs are updated when meaningful change is inferred whereas they remain stable in noisy environments. As mentioned, implementations of dynamic learning rates vary widely and we will draw comparisons amongst them in the following as well as in the discussion (there, with a stronger focus on the dissociation between noise and meaningful change). Also being error-update models, it may seem redundant to note that prediction errors are crucial in increasing the update weights in the HGF and CPD. This is similar in the presented versions of ‘model-free' learning, i.e. when no task structure is inferred. Here, high updates are operationalized via prediction errors affecting the learning rate, either via the previous trial's prediction error (12, 48), the current one (30) or the history of PEs reflected in their slope (32). As mentioned and in contrast to other similar models, the prediction error in the model by Stuke et al. (30) has an opposing effect on learning. In this task's stable environment, a high PE signals probabilistic noise and the individual tendency to downplay such information is captured in a free parameter. In relation to that and also working in a stable environment, the decay factor in Pearce Hall learning (12, 48) downregulates learning from information later in the task. In the adaptive model, this is complemented by the inverse precision directly based on the reward distribution of the task, and that further downregulates learning (48). Thereby, this task-informed trajectory serves a very similar function as the first-level uncertainty from the HGF, the noise parameter in the CPD and (inversely) the certainty trajectory of the model by Vinckier et al. (29). The latter model further describes a direct relationship between certainty driving more exploitative behavior, which is similar to the use of the 3rd level belief in the HGF as dynamic decision noise. Taken together, in our examples of non-hierarchical learning, there is only expected uncertainty driving learning up or down; learning rates are based on (1) the *Q*-values' optimality (29), (2) prediction errors (30, 32, 48) and/or (3) their (signaled) precision (48). In contrast, the CPD, HGF and HMMs further take into account hidden changes of the environment (unexpected uncertainty) and propose respective computational correlates. In the HMM, the transition rate parameter between task states is an equivalent to the meta-volatility of the HGF or the hazard rate in the CPD. In Bayesian inference, the recency bias for updating results in slower updating and lower learning rates (11). It might thereby serve a similar function as the decay factor (12) and though not being tested in a task with a changing environment (11), further points toward lower volatility/change point estimates.

## Discussion

In this review, we presented various studies that targeted the computational underpinnings of dynamic belief updating in psychosis. There was strong variety across tasks, models and patient groups. Tasks included reversal learning paradigms with varying difficulty (numbers of reversals and noise levels) that either used a social framing (23, 24, 26) or none (13, 21, 22, 25, 31). Further, there were versions of the beads task (11, 19, 30) as well as relevance inference in conditioning paradigms (18, 20) and tasks that required predicting the mean of numeric distributions (27, 28, 34). Moreover, within this wide range of tasks, very similar tasks were modeled in different frameworks, e.g. reversal learning tasks with the HGF [e.g., (21, 22)] or HMM [e.g., (13)] and numeric predictions with modified Pearce-Hall learning models (34) as well as with change point detection modeling (27, 28). Further, as we defined psychosis very broadly in our search scope, populations ranged from healthy subjects with some degree of self-reported delusional ideation (18, 25, 26, 30) over clinical high-risk patients (21, 34) to chronic schizophrenia patients (19, 20, 22, 27, 28, 31), with only few studies spanning transdiagnostic patient groups (19, 24, 25).

Given the high methodological and clinical heterogeneity, we aim to synthesize the psychosis finding on learning under noise and volatility across models and tasks. To achieve this, we will first compare how the different computational approaches model meaningful changes of the environment and how they deal with imprecise information and decision noise. Please note that we follow a solely descriptive approach here, for comparisons with a stronger emphasis on simulations see e.g. (36). Secondly, we will summarize the results across models and tasks and discuss their implications for our clinical understanding of psychosis. Lastly, we will end with selected aspects to be considered in future studies.

### Comparing the Different Modeling Approaches

#### Modeling Meaningful Changes in the Environment

All dynamic models implement the idea that when the environment changes learning should increase, whereas updates decrease over time when the environment remains stable. In the experimental paradigms this change is operationalized differently. For tasks with binary inputs and responses these changes are reflected in either reversals of the better option in reversal learning tasks [e.g. (13)] or similarly, in changes in trustworthiness of the advisor during advice (23). For the tasks using continuous inputs and responses, changes are operationalized via changes in the mean of the underlying distribution [e.g. (28)]. In line with these different task constructs, the computational correlates of meaningful change differ as well.

In the modified reinforcement learning models (32, 48) there is no belief trajectory explicitly tracking the environmental volatility. Instead, previous prediction errors are used to signal surprise and upregulate the current learning rate. In PH, a constant decay factor counterweights the effect of surprise and accounts for decreased updating during stable environmental conditions.

In both the CPD and the HGF as well as the confidence-learning model (29), dynamic “meta”-beliefs are learned that reflect the volatility of the environment. In the study by Vinckier et al. (29), this meta-belief reflects the subjective confidence or certainty that a respective choice leads to optimal outcomes and in states of high confidence, disconfirming evidence (i.e., high prediction errors of the opposing direction) are down-regulated and thus explained away as noise. Interindividual differences in how strongly confidence affects lower-level updates are reflected in a free weighting parameter γ. This is similar to the κ-parameter in the HGF that regulates how much higher-level beliefs affect the lower-level updates. In the HGF, the meta-belief reflects the environmental volatility belief and opposed to the Vinckier-model, higher-levels would affect higher updates for all lower-level prediction errors [as opposed to higher learning rates applied to only the disconfirming PEs at high uncertainty (29)]. Presumably, subjective confidence might be mirrored in the precision ratio, encompassing unexpected uncertainty about the environmental change as well as about expected uncertainty due to noisy (i.e. probabilistic) information. Thus, for stable but noisy environments, subjective confidence might be mirrored in first-level beliefs [probability estimates of the hidden state to be x, as implemented in (19)]. In dynamic environments, one could also interpret the HGF's volatility as the inverse subjective confidence and this becomes clearer when looking at the precision weights; the lower-level precision in the denominator of the update weight determining the learning rates encompasses the volatility belief (see **Equation 1**). However, this remains speculative, as explicit trial-by-trial confidence ratings have not been combined with this line of modeling [apart from a 2level-HGF (19)]. Interindividual differences in volatility learning are parametrized in the m3-parameter, as well as the initial starting point $\mu_{3}^{0}$ and ϑ, which represents the step-size of belief updating about volatility, sometimes also called ω_3_. The starting point seems to be crucial in determining the volatility level not only during the beginning but instead for the whole task, as can be seen by heightened μ_3_-levels across the whole task (22). The step-size parameter ϑ can be regarded as the “meta-volatility,” i.e. how quickly the volatility belief can change. In the HGF, the m3-parameter draws the volatility belief and thereby also the update to a certain equilibrium. Depending on the value of this equilibrium, this can have increasing (if the parameter is higher than the current belief) or decreasing (for lower equilibria) effects on the learning rate.

In CPD, the change point probability serves a very similar purpose to the volatility belief by upregulating the learning rate when change points are thought to be more likely than noisy inputs. The parameter informing the interval length between change points is the hazard rate. This parameter is usually fixed and close or equivalent to the ground truth in the task design. It can be compared to the transition rate in HMMs (also usually fixed), reflecting the likelihood of changes between states. In CPD, a decay of the learning rate is implemented via the dynamic trajectory of relative uncertainty that closely follows the change point probability. It is thus not simply dependent on time, but time in between change points.

#### Modeling Noisy Feedback

Tasks and models further differ in how they define and deal with meaningless or irrelevant noise in the sense of information-theoretic surprise (51–53). In binary task designs where subjects can choose between categories, noise is usually operationalized via probabilistic events, i.e. the better stimulus leading to loss or the worse one leading to reward in 10 percent of trials (in 0.9/0.1 contingencies). In prediction tasks (27, 28, 34) with continuous inputs and responses, noise is reflected in the variance of the underlying distribution. In the adaptive PH model (48), this variance (or standard deviation) is directly fed into the model weighting learning rates as well as prediction errors. Thereby, high noise leads to lower updates. In an equally stable, but binary task design where high prediction errors were only elicited by probabilistic events (30) the prediction error itself was used to directly downregulate the learning rate. The definition of noise in CPD is more comparable to adaptive PH model; it is a (again usually fixed) parameter σ_Noise_ that downregulates the relative uncertainty and thereby the learning rate. In the HGF, there is no clear-cut parameter or trajectory for noise. However, the dynamic uncertainty σ_2_ associated with the second-level belief reflects the *informational uncertainty*. It is lower the more the (stimulus-outcome-contingency) belief differs from 0.5. This is in contrast to CPD and adaptive PH, because this trajectory does not down-regulate the learning rate, but increases the precision ratio (equaling the learning rate) instead. In other words, the lower the precision of the belief the more needs to be learned. This is more in line with the operationalization by Vinckier et al. (29), where high confidence (being similar to precision) decreases learning, but here specifically from disconfirming prediction errors whereas it is universal in the HGF.

#### Decision Noise

Noise does not only play a role in terms of probabilistic and uninformative feedback, it is often also applied to describe unpredictable behavior, as in the term “decision noise.” The latter refers to stochasticity in choices, in the sense that participants do not follow the learned values. High decision noise can indicate that the wrong model is used, e.g. in case patients employ a simple heuristic to guide their decisions. Therefore, careful model comparison is warranted and differences in model fit between groups need to be considered. On the other hand, high decision noise can be seen as a tendency for exploration over exploitation of the acquired knowledge, i.e. learning trajectories like *Q*-values. In paradigms with binary choices, the beta temperature parameter, determining the steepness of the softmax function, reflects decision noise as a stable tendency across the task. Notably, some of the reviewed studies also propose a dynamic operationalization of noisy behavior depending on the beliefs on volatility (21–26) or confidence (29). This means that in states of high volatility or low confidence subjects would choose and *explore* the currently less optimal option more often. While information-seeking is undoubtedly useful in changing or uncertain environments, it is not the only reason for choice stochasticity that may also reflect noisy, less precise reward learning (54). The dissociation between both, exploration due to information seeking vs. learning noise, is non-trivial and requires more complex task designs than e.g. binary (in terms of inputs and responses) reversal tasks as mostly presented in this review (see Table 1). However, this constraint should be kept in mind when interpreting results on higher-order learning that are fitted on noisy choices, e.g. high switching rates in stable task phases.

### Synthesis of Psychosis Findings

Across most studies reviewed for this article, psychosis related to behavioral deficits in performing the experimental paradigms. As has been shown in the previous paragraphs, models differed in their mechanistic explanations. Despite the wide range in task designs and clinical statuses, the computational findings seem surprisingly in line; overall, in psychosis, there is a tendency to not stabilize beliefs but to constantly update them and thereby failing to reach an equilibrium in higher-level beliefs that would enable participants to downplay noise. Psychologically, this might lead to an experience of constantly increased alertness or uncertainty (not to be confounded with the computational definitions in the HGF or CPD). However, studies differ in where in the learning hierarchy this alteration may be located. In this second section of the discussion, we will first compare the findings on noise detection and higher-level learning in psychosis. We will then describe the computational mechanisms that were found to underlie these findings. Lastly, we will discuss the lack of relationships with clinical symptoms and the potential methodological pitfalls that may impede uncovering such correlations.

### Over-interpreting Noise as Environmental Change Causing Instable Beliefs

A long and prominent tradition in schizophrenia research has highlighted the role of insufficient down-regulation of irrelevant information in the formation of psychotic symptoms (4–8, 40). In computational approaches this may find its correlates in high prediction errors elicited by probabilistic events in stable environments (19, 30), task-irrelevant information (18, 20) or less precise inputs (27, 28, 34). Nevertheless, this might be partly due to high cognitive task demands, since the noisiness of the environment had to be indirectly inferred in most paradigms. However, even when the variance of inputs was explicitly cued, patients still did not adapt their belief update accordingly. Adams et al. (19) proposed an attractor-like mechanism leading to unstable beliefs with larger updates toward unexpected evidence and smaller updates to expected evidence. This is in line with the observation by Nassar et al. (28) that learning rates were either very high or very low in PSZ. Both, no precision weighting and a bias for disconfirming events, were observed in noisy, but stable environments where higher-level beliefs were not taken into account (19, 34).

In theory, when irrelevant noise is given too much weight, the associations cannot approximate to an equilibrium and thereby, higher-level beliefs on volatility may increase while estimated confidence would remain low. This is in line with many of the studies that manipulated this higher-order context in psychosis: change point probabilities [(28), but see (27)] and volatility estimates were increased (21, 22, 25, 26) and under ketamine serving as a model for psychosis, the effects of confidence were decreased (29). Alternatively to the idea that the over-interpretation of lower-level noise prevents any sense of stability, these latter findings may also suggest that the environment seems to be constantly changing to the psychotic mind causing hyper-updating of beliefs. Though we doubt that the causal direction of these phenomena can be disentangled with the given paradigms (please see the next section for a methodological discussion), these findings overall indicate a state of increased uncertainty in psychosis. It should however be noted that the findings by Nassar et al. (28) draw a more complex picture; learning rates were not constantly high, but there were also trials of perseveration (“all or nothing”). And though it seems functional to rapidly adapt behavior in states of high volatility, this does not seem to pay off in psychosis; compared to controls, schizophrenia patients still showed worse performance levels when only taking into account the *ground truth* volatile tasks phases [(22, 24); no equivalent raw data analyses reported by the other studies].

### The Computational Parameters Altered in Psychosis

A variety of computational mechanisms might underlie the increased uncertainty attributed to the environment in psychosis. While learning parameters were not altered in the CPD studies (also due to a different modeling approach, see next section), HGF parameters were found to differ in psychosis (see Table 1). Here, increased volatility estimates were related to increased parameters for the mean reverting equilibrium (21), the initial volatility belief (22, 25, 26) and the coupling between higher and lower-level beliefs belief (22, 25, 26). Interestingly, the latter parameter κ was not found to be increased in CHR (21). However, this difference cannot be easily attributed to symptom severity or clinical state since κ was also increased in a largely healthy sample with subclinical delusional ideations (25). Thus, a methodological reason seems more likely. Unlike in the other reversal studies (22, 25, 26), the volatile and thus more difficult phase containing several reversals came last in the task used by Cole et al. (21). Therefore, one might speculate that in the other studies, subjects did not “recover” in their performance when phases with more reversals or more difficult contingencies were followed by easier or more stable phases. This once adaptive increase in uncertainty about the environment could not be accordingly reversed, which might be captured by the increased κ parameter. But note that findings in delusion-proneness were similar though less pronounced in an alternate version of the task beginning with the more difficult contingencies (25). However, such heightened volatility beliefs were not due to decreased meta-volatility (i.e. slower learning about volatility), as only Suthaharan et al. (26) reported lower ϑ values in delusion proneness and there also seem to be methodological difficulties concerning the fitting and thus specific interpretability of this parameter. Taken together, these findings point toward a biased tracking of only high volatility. Context-independent over-estimation of volatility in psychosis might still allow for rapid updating in truly volatile environments. But crucially, when the environment turns stable again this does not *re-stabilize* the beliefs. In line with this notion of reactance, a different modeling approach revealed that patients relied more on prior information gathered earlier in the task when environments remained stable (11).

### The Relationship Between Belief Updating and Symptom Domains

In contrast to the reviewed findings of higher volatility beliefs in psychosis, clinical and subclinical hallucinations were accompanied by decreased phasic volatility signatures when inputs encompassed perceptual ambiguity (55, 56). According to the hypothesis, hallucinations result from an overreliance of perceptual beliefs over sensory evidence (57, 58). In other words, when a subject has built the expectation that a stimulus appears in a specific context, this prior belief may bias perception. This was investigated by first inducing Pavlovian learning between a visual target and a co-occurring tone. In the task itself, the intensity as well as the occurrence of the tone was gradually decreased. In line with the hypothesis, clinical and non-clinical voice-hearers (55), as well as CHR (56) overestimated the occurrence of the tone. Computationally, this was reflected in a higher weighting of prior beliefs over the sensory input (55, 56). In a different task design with ambiguous visual stimuli, delusion-prone subjects were more susceptible to social information that biased perception away from current sensory inputs [(59); also see next paragraph]. These findings in the perceptual domain suggest different alterations in dynamic belief updating between perceptual and higher-cognitive systems in the development of psychotic symptoms [e.g. (10)]. To delineate those, tasks targeting the perceptual and the higher-cognitive domain need to be assessed in the same participants.

Crucially, the specificity of higher-level learning deficits to symptoms of psychosis remains largely unclear. Theoretical accounts have proposed a link between aberrant learning due to increased endogenous subjective uncertainty [e.g., (9, 60)] and delusions, specifically. And etiologically, reacting to changing environments with heightened and uncorrectable uncertainty might relate to an upregulation of mistrust following traumatic experiences (61). Indeed, many of the group differences in the studies reviewed here point toward instable beliefs being caused by higher-level beliefs about environmental uncertainty (encompassing change points and volatility beliefs). However, the correlational evidence for such deficit being involved in the formation of clinical symptoms of psychosis, or delusions in particular, is still lacking (see our Table 1). Instead, computational parameters often related to measures of general cognition (19, 22, 28). It has further been shown that working memory greatly contributes to reinforcement learning (62) and in tasks differentiating this from incremental belief updating, schizophrenia patients showed deficits in the working memory domain only (63). Nevertheless, this does not exclude a relationship between higher level learning deficits and psychotic symptoms. Instead, the reviewed experimental paradigms were often transferred without many changes from cognitive neuroscience studies designed for healthy controls [e.g., (32, 64, 65)]. These often seem to be too difficult and demanding for patients as can be seen in the lower raw data performance and in the use of less complex models not sufficing the task structure (13, 34). Such floor effects might prevent the uncovering of presumably more fine-grained psychosis related behavioral alterations beyond decision noise. In terms of modeling, this might further be complicated via the conflation between the concepts of high volatility, decision noise and information seeking. However, it should be noted that one study that addressed such methodological issues elegantly found a different pattern in the beads paradigm. When controlling for cognition, socio-economic status and task comprehension, delusions were specifically correlated with increased reliance on prior beliefs resulting in more information seeking (11). Crucially, this delusion-specific belief updating alteration was at odds with the pattern in the overall group of schizophrenia patients who seemed to jump to conclusions via decreased information seeking. Further studies applying such rigor in terms of patients' characterization and task design are warranted. Until then, we can only conclude that (1) schizophrenia patients and delusion prone subjects overall show instable beliefs mechanistically explained by increased uncertainty estimation and (2) that relying too much on prior information reflects the mechanism specific to (sticking to) delusions.

Further, the use of stimuli inherent to typical psychosis topics remains a promising approach (23, 24). Psychotic reasoning often revolves around social topics as seen in persecution and hostility by others (66). In their review on uncertainty and delusions, Feeney et al. (60) propose that this might be due to particular deficits high in the learning hierarchy, as belief updating about social content is more complex. So far, studies taking on this challenge of social framings on learning along the psychosis continuum have produced a rather diverse picture; ranging from no differential effects on learning (26) over decreased susceptibility to any social information (23), to increased learning from social cues (24). The variance in findings might be affected by differences in contingencies of the associations (and the respective uncertainty) in the tasks. As has been shown by Rossi-Goldthorpe et al. (59) in the field of perception, higher susceptibility to positive *and* negative social cues was observed to be more pronounced in states of higher uncertainty about one's own perception as well as when these social beliefs were more uncertain (as in 0.5 compared to 0.75 probability of the social prompt to be either misleading or helpful). Future studies should carefully disentangle, whether this tendency for self-deception also holds for higher-level beliefs as for associations between neutral events and hostile interpretation, as in our example from the introduction where the neighbors' behavior is tracked and over-interpreted. As the data by Rossi-Goldthorpe et al. (59) suggest for perception, increased uncertainty serves as the prerequisite for social susceptibility. It remains to be disentangled for the area of belief updating, whether increased uncertainty is (at least initially) causal in driving attention to social but unreliable events, while a reciprocal interaction might also be clinically reasonable.

### Outlook

As reviewed above, alterations in dynamic belief updating are at the core of the development and maintenance of psychotic symptoms. Computational methods provide powerful tools to investigate the underlying mechanisms and already some consistent findings emerged. Based on the reviewed studies, the following aspects should be considered regarding task design, modeling, and participants.

#### Task Designs: Not Too Complex but Maximally Informative

Behavioral tasks should be carefully designed in order to achieve the challenging task of balancing clinical applicability and maximally informative readouts. In other words, participants, and among them patients with cognitive deficits, should be able to perform these tasks while the behavioral readouts should be well-suited to dissociate between updating too much or just performing noisily. The latter could be achieved by experimentally manipulating the level of volatility and noise in separate conditions. In addition, the use of continuous instead of binary inputs and responses may be better suited for differentiating volatility and noise. Cognitive demands should be minimized as far as possible for participants, especially in patient populations who may be affected by cognitive impairment or symptom burden. Important measures such as prediction errors could explicitly be shown in the paradigm [e.g. as in the paradigms by Nassar et al. (16) or Diederen and Schultz (48)], instead of having to infer these as hidden states by using computational modeling. The addition of trial-wise confidence estimates can improve fitting of higher-level beliefs (67); however, such modifications increase the cognitive demands and task lengths and might not be suitable for all patient samples. Another important issue of task design relates to the kind of stimuli used and their social and affective importance to the participants. As mentioned above psychotic experiences often center around social situations and emotions seem to be highly relevant for the emergence of delusion formation (68). Therefore, it might be promising to explore paradigms of belief formation using affectively relevant stimuli or ideally to assess the belief updating mechanism in an affectively neutral and emotional relevant context.

#### Modeling: Applying the Gold Standard

Regarding behavioral computational modeling, there are excellent suggestions and tutorials for establishing a gold standard (17). These have yet to be meet by clinical studies. For example, simulation and parameter recovery, already assessed by many of the studies (see Table 1), should be used to show that certain model parameters indeed explain observable behavior. In general, identifiability may be hindered when using very complex models and incorporate many free parameters. Regarding clinical studies, two interconnected aspects seem especially important: poor task performance and the use of different models by different participants. Poor task performance (chance level performance) can result in high decision noise and may impede model identifiability and interpretability of model parameters. Furthermore, different strategies may be used by individual participants resulting in different models explaining the observed behavior of participants best. The latter can be identified with model comparisons on the group and individual level, but there is yet no established standard to deal with those issues. Participants can be excluded due to performance cut-offs or poor model fit, but this limits the generalizability and may result in excluding the more severely affected patients. On the other hand, comparability of parameters across participants may be compromised if models are not identifiable due to chance level behavior or if different models were used by participants. One approach may be to use nested models, where the different strategies are incorporated into one model and the individual degree of using a particular strategy is expressed by a free parameter (31, 69). Another issue concerns fitting procedures. In all accounts but the CPD (28) parameters of interest that “act” directly on the learning or decision process are freely fitted. This might be the more intuitive option, since interindividual differences directly appear at the mechanisms of interest. Apart from decision noise however, the values of these parameters are usually not directly (anti)proportional to optimality; especially when there is a variety of free parameters, different constellations of parameters can explain very similar responses (70). Therefore, simulations are warranted to control for parameter and model recovery [(17, 70); see Table 1]. In contrast, the CPD uses normative modeling (28). Optimal learning is simulated based on parameters (such as the hazard rate or noise) that resemble that actual task environment. These optimal trajectories are then used as predictors for behavior, so that results refer to the optimality degree.

#### Sample: Psychosis Spectrum

Participants across the psychosis spectrum should be investigated with similar tasks and computational models, ideally in transdiagnostic and longitudinal studies. This would allow us to investigate if changes in computational parameters depend on disease states (CHR, FEP, chronic) and to assess specific associations with psychopathology (delusions, hallucinations, and negative symptoms and their differentiation from neurocognitive impairments). Importantly, careful control analyses for general cognitive abilities, socio-economic status and task comprehension, are warranted to avoid misleading results (11). To achieve this, collaborative efforts are necessary. Tasks and scripts should be public to help other researchers to apply the exact same task with the same instructions and modeling approach, in order to compare data across sites.

## Conclusion

Taken together, our review showed that different methodological approaches converge on the finding that psychotic participants tend to overestimate the changes in the environment related to a state of high uncertainty about the task-adaptive responses. To further investigate the exact nature of this alterations, reliable tasks across participants from the psychosis spectrum in combination with meeting high methodological standards on the computational modeling side are warranted. Such a computational psychiatry approach holds the promise to advance our mechanistic insight into the cognitive anatomy of psychotic experience.

## Author Contributions

TK and FS: conception of the work. TK, SF, and LW: literature research. TK, SF, LW, and FS: drafting the article. All authors contributed to the article and approved the submitted version.

## Funding

This work was supported by the German Research Foundation (grant number SCHL 1969/1-2/3-1/5-1) and by the Einstein Center for Neurosciences (LW and SF).

## Conflict of Interest

The authors declare that the research was conducted in the absence of any commercial or financial relationships that could be construed as a potential conflict of interest.

## Publisher's Note

All claims expressed in this article are solely those of the authors and do not necessarily represent those of their affiliated organizations, or those of the publisher, the editors and the reviewers. Any product that may be evaluated in this article, or claim that may be made by its manufacturer, is not guaranteed or endorsed by the publisher.
